# Retraction: Intermittent Hypoxia Effect on Osteoclastogenesis Stimulated by Neuroblastoma Cells

**DOI:** 10.1371/journal.pone.0198763

**Published:** 2018-06-05

**Authors:** The PLOS ONE Editors

The authors of this article [1] notified the journal office that the University of Illinois at Chicago had investigated this work and that concerns had been raised about results presented in Figures 4, 5, and 6.

Figure 4B: The image in row 3, column 2 (SP600125, 5.0 μM), was duplicated in row 4, column 1 (SB203580, 0.1 μM). This duplicate image was replaced in a Correction [2] that was published before the journal office was notified of the institution’s investigation.

Figure 5A: Similarities were noted between the following image pairs:

- row 1 column 1 (N (0 h), bottom portion) and row 2 column 3 (IH siHIF-2α (12h), top portion)
- row 1 column 4 (IH (12h), top portion) and row 2 column 1 (IH + siNTC (12h), bottom portion)

Figure 6A: The same mouse appears to be presented in the third and fourth images of the left-hand panel (Parental, N).

The original data underlying these results are not available. The University of Illinois at Chicago advised that per their investigation the reliability of data and results presented in this article have been called into question.

In light of these issues, and in line with the University of Illinois at Chicago’s recommendation, the authors and *PLOS ONE* Editors retract this article.

BVK, MI, GM, MS did not respond to this retraction.
